# Clinical features and prognosis of systemic lupus erythematosus complicated by active cytomegalovirus infection: a retrospective cohort study

**DOI:** 10.3389/fimmu.2024.1323923

**Published:** 2024-02-28

**Authors:** Yan Chen, Lifan Zhang, Yuchen Liu, Ye Liu, Lidan Zhao, Baotong Zhou, Guiren Ruan, Xiaochun Shi, Xiaoqing Liu

**Affiliations:** ^1^ Division of Infectious Diseases, Department of Internal Medicine, State Key Laboratory of Complex Severe and Rare Disease, Peking Union Medical College Hospital, Chinese Academy of Medical Sciences and Peking Union Medical College, Beijing, China; ^2^ Center for Tuberculosis Research, Chinese Academy of Medical Sciences and Peking Union Medical College, Beijing, China; ^3^ Clinical Epidemiology Unit, Peking Union Medical College, International Clinical Epidemiology Network, Beijing, China; ^4^ Department of Rheumatology and Clinical Immunology, Clinical Immunology Center, Chinese Academy of Medical Sciences and Peking Union Medical College, Beijing, China

**Keywords:** systemic lupus erythematosus, cytomegalovirus, organ involvement, mortality, recurrence

## Abstract

**Objective:**

The aim of this study was to investigate the clinical traits and consequences of systemic lupus erythematosus (SLE) complicated by active cytomegalovirus (CMV) infection.

**Methods:**

This retrospective review involved the examination of medical records for patients diagnosed with SLE who had an active CMV infection at the time of their discharge from Peking Union Medical College Hospital between June 2016 and December 2022. The consistency between plasma CMV deoxyribonucleic acid (DNA) viral load and pp65 antigenemia was analyzed using the chi-square test. Related factors for CMV disease in SLE complicated by active CMV infection patients were analyzed by univariate analysis and multivariable stepwise logistic regression. Cox hazards regression analysis was used to determine predictors for all-cause mortality and CMV recurrence within 3 months.

**Results:**

A total of 206 patients were enrolled in this study. Of the 123 patients who were detected with both plasma CMV DNA viral load and pp65 antigenemia within an interval not exceeding 72 h, the consistency between plasma CMV DNA viral load and pp65 antigenemia was not good (Kappa = −0.304, *p* < 0.001). Plasma CMV DNA viral load ≥ 1,600 copies/mL [odds ratio (OR) 4.411, 95% CI 1.871–10.402, *p* = 0.001], current glucocorticoids dose (equivalent to prednisolone) ≥60 mg/d (OR 2.155, 95% CI 1.071–4.334, *p* = 0.031), and elevated alanine transaminase (OR 3.409, 95% CI 1.563–7.435, *p* = 0.002) were significant clinical clues indicating CMV disease in SLE. Multivariable Cox hazards regression analysis showed that CMV organ involvement [hazard ratio (HR) 47.222, 95% CI 5.621–396.689, *p* < 0.001], SLE multi-system involvement (HR 1.794, 95% CI 1.029–3.128, *p* = 0.039), and elevated hypersensitive C-reactive protein (hsCRP) (HR 5.767, 95% CI 1.190–27.943, *p* = 0.030) were independent risk factors for 3-month all-cause mortality. CMV organ involvement (HR 3.404, 95% CI 1.074–10.793, *p* = 0.037) was an independent risk factor for CMV recurrence within 3 months.

**Conclusion:**

In SLE patients, plasma CMV DNA viral load seemed to have a higher value in the diagnosis of CMV disease; patients with CMV organ involvement, SLE multi-system involvement, and elevated hsCRP might have a higher risk of 3-month all-cause mortality; and patients with CMV organ involvement might have a higher risk of CMV recurrence within 3 months.

## Introduction

Cytomegalovirus (CMV), a double-stranded deoxyribonucleic acid (DNA) virus, is known to establish a state of lifelong latency following infection (1, 2). In China, seroprevalence of adult CMV has been reported to be as high as 90% (3). Under specific circumstances, CMV can be reactivated, leading to recurrent infections. Typically, CMV infection is either symptomless or manifests as mononucleosis-like symptoms, including fever, sore throat, and fatigue. Nevertheless, CMV harbors the potential to inflict damage upon diverse organs, including the lungs, liver, gastrointestinal tract, central nervous system, and retina, consequently amplifying the mortality risk for immunocompromised individuals (4–6). The most common methods of CMV infection diagnosis are plasma CMV DNA viral load and pp65 antigenemia test. The reliability of CMV infection diagnostic test raises questions due to the variability introduced by factors such as sample stability and leukopenia.

The prevalence of systemic lupus erythematosus (SLE) in China ranges from approximately 30/100,000 to 70/100,000 (7). Patients who received glucocorticoids (GCs) (equivalent PSL dose ≥20 mg/d, plus treatment course ≥14 days, or total equivalent PSL dose >700 mg), biological agents, or immunosuppressants are considered in an immunosuppressive state (8). Infection is the leading cause of death in SLE patients; SLE patients who received immunosuppressive treatment are at increased risk of infection, and the identification and prevention of infections, such as active CMV infection, should be strengthened (9). When SLE patients develop fever or other clinical symptoms suspected of active CMV infection, CMV infection should also be considered and actively tested. Previous studies have reported that the prevalence of CMV antigenemia was 35.1% in whole patients with autoimmune diseases, up to 58.6% in patients with SLE, and 11.4% in patients with non-SLE autoimmune diseases (10). In another single-center study, CMV DNA was detected in 17.0% (142/834) of patients who received corticosteroid therapy for RD (11). The coexistence of CMV infection and SLE requires many complex considerations. Active CMV infection can mimic clinical manifestations of SLE (12, 13) and treatment options for SLE patients may be limited because of serious infection. On the other hand, CMV plays a potential role in triggering SLE and SLE disease activity (14, 15). It is thus important to study CMV infection in SLE patients.

Although some scholars have conducted active exploration in the clinical significance of CMV infection in human immunodeficiency virus (HIV)-infected patients and transplant recipients, there is still a lack of understanding of active CMV infection in SLE patients. This study endeavors to summarize the characteristics and outcomes of SLE patients complicated by active CMV infection, investigate the association and diagnostic efficacy of plasma CMV DNA viral load and pp65 antigenemia, and explore risk factors for all-cause mortality and CMV recurrence within 3 months. This work is valuable for the management of CMV infection in SLE population.

## Materials and methods

### Study design and patients

This study follows a retrospective cohort design, in which we included SLE patients who were hospitalized and had active CMV infection at Peking Union Medical College Hospital between June 2016 and December 2022. Inclusion criteria were as follows: (1) age 16 years or older; (2) meeting the 2009 American College of Rheumatology (ACR) SLE classification criteria (16); and (3) satisfying the diagnostic criteria for active CMV infection. Exclusion criteria were as follows: (1) cases with an overlap syndrome (where SLE overlaps with other rheumatic diseases); and (2) transplantation or other non-SLE immunocompromising conditions.

We collected data on patient demographics; disease progression; the SLE disease activity index (SLEDAI)-2000 (17); medication usage including GCs, immunosuppressants, and biological agents; plasma CMV DNA viral load; and pp65 antigenemia, as well as laboratory findings such as routine blood tests. All laboratory analyses were performed by the Laboratory Department of Peking Union Medical College Hospital. This study received approval from the institutional ethics committee at Peking Union Medical College Hospital (No. I-23PJ1192).

### Laboratory testing

Plasma CMV DNA viral load: The CMV DNA diagnostic blood kit (Sansure Biotech, China) with a limit of detection of 500 copies/ml was performed to extract DNA extraction of the plasma sample. A real-time fluorescent quantitative polymerase chain reaction (PCR) detection of CMV DNA was performed on a LightCycler 480 Detection System (Roche, US) using Therma-Base Taqman technologies.

CMV pp65 antigenemia: The CMV Brite assay (IQ products BV, Groningen, the Netherlands) was performed as instructed. EDTA anti-coagulated whole blood samples (for patients with neutropenia, at least 5–6 ml of blood was drawn) were processed and approximately 1.5×10^5^/0.1 mL cells were immunofluorescence stained with anti-CMV pp65 antibodies (C10/C11 and IgG1) after fixation. Then, they were re-stained with FITC-labeled rabbit anti-mouse IgG conjugate. The slides were read using a fluorescence microscope (Olympus BX51, Tokyo, Japan). Polylobate perinuclear yellow-green fluorescent staining of leukocytes was used to determine positive CMV antigenemia. One or more CMV antigen-positive cells present per duplicate stain was considered positive.

### Definition

Active CMV infection: presence of CMV replication in tissue, blood, or other bodily fluids regardless of symptomatology. CMV replication is detected by nucleic acid testing, antigen testing, H&E stain, and/or immunohistochemistry. Active infections were classified as subclinical CMV infection or CMV disease based on the presence or absence of symptoms related to CMV.

Subclinical CMV infection: CMV replication without clinical signs and symptoms of disease.

CMV disease: CMV infection that is accompanied by clinical signs and symptoms, including CMV syndrome (fever, malaise, atypical lymphocytosis, leukopenia or neutropenia, and thrombocytopenia) and CMV organ involvement (e.g., pneumonia, hepatitis, gastrointestinal disease, and retinitis) (**Table 1**). The diagnosis of CMV infection/disease was independently established by two infectious disease specialists, with any disagreements resolved through consultation with a third senior physician.

**Table 1:** Categorization of active CMV infection.

Diagnostic category		Criteria
Subclinical CMV infection		CMV replication without clinical signs and symptoms of disease.
CMV disease	CMV syndrome	At least two of the following: Fever ≥ 38°C for at least 2 d; new or increased malaise or fatigue; leukopenia or neutropenia on two separate measurements; 5% atypical lymphocytes; thrombocytopenia.
	CMV pneumonia	Clinical symptoms and/or signs of pneumonia such as new infiltrates on imaging, hypoxia, tachypnea, and/or dyspnea plus the detection of CMV in BALF or the absence of other documented cause of pneumonia.
	CMV hepatitis	Abnormal liver tests without other documented cause of hepatitis.
	Gastrointestinal CMV disease	Presence of upper and/or lower GI symptoms plus macroscopic mucosal lesions plus CMV documented in tissue; patients can be clinically diagnosed upon fulfillment of typical GI symptoms plus the absence of other potential causes plus effective response to antiviral treatment.
	CMV retinitis	Typical ophthalmological signs as assessed by an ophthalmologist experienced with the diagnosis of CMV retinitis.

CMV, cytomegalovirus; GI, gastrointestinal.

CMV recurrence: plasma CMV DNA viral load or pp65 antigenemia turned positive again within a temporal window spanning from 7 days to 3 months regardless of symptomatology.

### Statistical analysis

Normally distributed variables are typically represented as means with a standard deviation (SD), while non-normally distributed variables are often indicated by the median and interquartile range (IQR). Categorical variables are usually expressed as percentages (%). To compare continuous variables that follow a normal distribution, the group *t*-test was employed, while for continuous variables that do not conform to normal distribution, the Wilcoxon test was used. When comparing categorical data between groups, the chi-square test or Fisher’s exact test was utilized. We used the chi-square test to assess the consistency between plasma CMV DNA viral load and pp65 antigenemia. Univariate and multivariable stepwise logistic regression analyses were employed to investigate factors related to CMV disease in SLE patients complicated by active CMV infection. Predictors of all-cause mortality and CMV recurrence within 3 months were identified through Cox hazards regression analysis. Statistical significance was determined for *p*-values < 0.05. Data analysis was carried out using Statistical Package for Social Sciences (SPSS) software version 26 and R programming software.

## Results

### Demographic and clinical characteristics

A total of 206 patients with SLE were enrolled in the study. In the overall study population, the average age of participants was 34 ± 14 years; 88.3% were women. In terms of SLE system involvement, lupus nephritis was the most common (160/206, 77.7%), followed by hematologic involvement (125/206, 60.7%), neuropsychiatric lupus (62/206, 30.1%), and serositis (61/206, 29.6%). Of the 206 patients enrolled, 204 patients (99.0%) were treated with GCs within 1 month, 70 patients (34.0%) received GCs pulse therapy (equivalent to methylprednisolone 500/1,000 mg/d), 164 patients (79.6%) were treated with immunosuppressants within 3 months [including cyclophosphamide (CTX), methotrexate (MTX), mycophenolate mofetil (MMF), azathioprine (AZA), cyclosporine A (CsA), leflunomide (LEF), and tacrolimus (FK506)], and 10 patients (4.9%) were treated with biological agents within 3 months (including rituximab and belimumab). Among the patients enrolled, 191 (92.7%) received antiviral therapy, and 15 (7.3%) have not been treated with antiviral therapy.

Of the 206 patients enrolled, 114 (55.3%) were positive for plasma CMV DNA viral load and 131 (64.2%) were positive for pp65 antigen. The 206 patients were grouped into the subclinical CMV infection group (*n* = 117) and the CMV disease group (*n* = 89) according to the definition above. The latter included CMV syndrome (56/89, 62.9%), CMV pneumonia (17/89, 19.1%), CMV hepatitis (13/89, 14.6%), CMV gastroenteritis (2/89, 2.2%), and retinitis (1/89, 1.1%). As shown in **Table 2**, compared with the subclinical CMV infection group, the proportion of male (*p* = 0.004), positive CMV DNA (*p* = 0.016), plasma CMV DNA viral load ≥ 1,600 copies/mL (*p* < 0.001), and received anti-CMV therapy (*p* = 0.003) were higher in the CMV disease group, and their hospital stays (*p* < 0.001) and course of antiviral treatment (*p* = 0.013) tended to be longer. The number of SLE involved system (*p* = 0.007) and current GCs dose [equivalent to prednisolone (PSL)] (*p* = 0.014) were significantly higher than in the subclinical CMV infection group. No significant differences were observed between the two groups in terms of SLEDAI score, GCs pulse therapy within 1 month, and immunosuppressive or biological agent use within 3 months. Antivirals were started pre-emptively in 103 (88.0%) patients without clinical symptoms of active CMV infection.

**Table 2:** General characteristics of 206 enrolled patients.

	Total (*n* = 206)	Subclinical CMV infection (*n* = 117)	CMV disease (*n* = 89)	*p*-value^a^
Male, *n* (%)	24 (11.7%)	7 (6.0%)	17 (19.1%)	0.004
Age (year, mean ± SD)	34 ± 14	33 ± 14	35 ± 13	0.433
Length of hospitalization stay (day, median, IQR)	29 (21, 45)	26 (20, 35)	39 (24, 53)	< 0.001
SLE duration^b^ (months)	30 (4, 107)	34 (5, 102)	25 (2, 108)	0.321
CMV DNA (+), *n* (%)	114 (55.3%)	56 (48.3%)	58 (65.2%)	0.016
CMV DNA (copies/mL)				0.006
<500	91 (44.2%)	60 (51.7%)	31 (34.8%)	
500–799	28 (13.6%)	19 (16.4%)	9 (10.1%)	
800–1,599	27 (13.1%)	14 (12.1%)	13 (14.6%)	
≥1,600	59 (28.6%)	23 (19.8%)	36 (40.4%)	
pp65 (+), *n* (%)	131 (64.2%)	80 (81.6%)	51 (68.9%)	0.053
Antiviral treatment, *n* (%)	191 (92.7%)	103 (88.0%)	88 (98.9%)	0.003
Course of antiviral treatment^c^ (day, median, IQR)	21 (14, 25)	21 (14, 21)	21 (16, 28)	0.013
SLEDAI-2000 (median, IQR)	13 (9, 18)	14 (9, 18)	12 (9, 17)	0.630
SLE involved system, *n* (%)				
Lupus nephritis	160 (77.7%)	89 (76.1%)	71 (79.8%)	0.527
Hematologic involvement	125 (60.7%)	60 (51.3%)	65 (73%)	0.002
Neuropsychiatric lupus	62 (30.1%)	36 (30.8%)	26 (29.2%)	0.809
Serositis	61 (29.6%)	30 (25.6%)	31 (34.8%)	0.152
Myocardial involvement	35 (17.0%)	16 (13.7%)	19 (21.3%)	0.146
Vascular involvement	34 (16.5%)	18 (15.4%)	16 (18%)	0.619
Respiratory involvement	31 (15.0%)	17 (14.5%)	14 (15.7%)	0.811
Gastrointestinal involvement	29 (14.1%)	16 (13.7%)	13 (14.6%)	0.849
Number of SLE involved system (median, IQR)	3 (2, 4)	2 (1, 3)	3 (2, 4)	0.007
Use of GCs pulse therapy^d^ within 1 month, *n* (%)	70 (34.0%)	39 (33.3%)	31 (34.8%)	0.822
Current GCs dose^e^ (mg/d, median, IQR)	55 (50, 75)	50 (45, 60)	60 (50, 80)	0.014
Use of immunosuppressant^f^ within 3 months, *n* (%)	164 (79.6%)	91 (77.8%)	73 (82%)	0.454
Use of biological agents^g^ within 3 months, *n* (%)	10 (4.9%)	6 (5.1%)	4 (4.5%)	1.000

a Subclinical CMV infection group vs. CMV disease group.

b SLE duration, the interval between initial SLE symptom onset and the current diagnosis of active CMV infection.

c Course of antiviral treatment, overall anti-CMV treatment course during this active CMV infection.

d GCs pulse therapy, equivalent to methylprednisolone 500/1,000 mg/d.

e Current GCs dose, equivalent to prednisone.

f Including any one of the following immunosuppressants: cyclophosphamide, mycophenolate mofetil, methotrexate, azathioprine, leflunomide, cyclosporine A, and tacrolimus.

g Including any one of the following biological agents: rituximab and belimumab.

CMV, cytomegalovirus; DNA, deoxyribonucleic acid; SLE, systemic lupus erythematosus; SLEDAI, SLE disease activity index; GCs, glucocorticoids.

### Consistency analysis of plasma CMV DNA viral load and pp65 antigenemia

Of the 123 SLE patients who were detected with both plasma CMV DNA viral load and pp65 antigen within an interval not exceeding 72 h and no treatment adjustments within the interval, 44 (35.8%) were positive for CMV DNA viral load, 104 (84.6%) were positive for pp65 antigen, 26 (21.1%) were positive for both CMV DNA viral load and pp65 antigen, and 1 was negative for both CMV DNA viral load and pp65 antigen (proven by the detection of CMV DNA in the bronchoalveolar lavage, compatible clinical and radiographic findings and effective antiviral response). There was a lack of consistency between plasma CMV DNA viral load and pp65 antigenemia (**Table 3**).

**Table 3:** Consistency of plasma CMV DNA viral load and level of pp65 antigen.

Groups	Items	pp65 (+)	pp65 (−)	Total	Kappa	*p*-value
Active CMV infection	DNA (+)	26	18	44		
	DNA (−)	78	1	79		
	Total	104	19	123	−0.304	<0.001
Subclinical CMV infection	DNA (+)	13	8	21		
	DNA (−)	52	0	52		
	Total	65	8	73	−0.234	<0.001
CMV disease	DNA (+)	13	10	23		
	DNA (−)	26	1	27		
	Total	39	11	50	−0.378	0.001

CMV, cytomegalovirus; DNA, deoxyribonucleic acid.

### Related factors for CMV disease in SLE complicated by active CMV infection patients

Related factors for CMV disease were analyzed in 206 SLE patients. The univariate analysis findings can be seen in **Table 4**. Subsequently, variables such as gender, CMV DNA viral load, pp65 antigenemia, number of SLE involved system, current GCs dose ≥ 60 mg/d, alanine transaminase (ALT), and hypersensitive C-reactive protein (hsCRP) were included in a multivariable logistic regression model. The finding indicated that plasma CMV DNA viral load ≥ 1,600 copies/mL (OR 4.411, 95% CI 1.871–10.402, *p* = 0.001), current glucocorticoids dose (equivalent to PSL) ≥ 60 mg/d (OR 2.155, 95% CI 1.071–4.334, *p* = 0.031), and elevated ALT (OR 3.409, 95% CI 1.563–7.435, *p* = 0.002) were independent related factors for CMV disease.

**Table 4:** Related factors for CMV disease in SLE complicated by active CMV infection patients.

Variable	Univariate analysis		Multivariable analysis	
	Odds ratio (95% CI)	*p*-value	Odds ratio (95% CI)	*p*-value
Gender				
Female	1		1	
Male	3.710 (1.465–9.394)	0.006	2.560 (0.900–7.278)	0.078
Age (years)	1.008 (0.988–1.028)	0.431		
CMV DNA (copies/mL)		0.007		0.007
<500	1		1	
500–799	0.917 (0.371–2.264)	0.851	1.068 (0.368–3.098)	0.904
800–1,599	1.797 (0.753–4.292)	0.187	1.536 (0.546–4.317)	0.416
≥1,600	3.029 (1.536–5.977)	0.001	4.411 (1.871–10.402)	0.001
pp65				
Negative	1			
Positive	0.499 (0.245–1.015)	0.055		
Number of SLE involved system	1.241 (1.021–1.509)	0.030		
SLEDAI-2000	0.983 (0.944–1.025)	0.429		
Use of GCs pulse therapy^#^ within 1 month				
No	1			
Yes	1.069 (0.598–1.912)	0.822		
Current GCs dose^$^ (mg/d)				
0–60	1		1	
≥60	1.929 (1.103–3.373)	0.021	2.155 (1.071–4.334)	0.031
Use of immunosuppressant^&^ within 3 months				
No	1			
Yes	1.304 (0.651–2.611)	0.455		
Use of biological agents^※^ within 3 months				
No	1			
Yes	0.871 (0.238–3.183)	0.834		
Pancytopenia				
No	1			
Yes	2.053 (0.835–5.044)	0.117		
ALT				
≤ULN	1		1	
>ULN	3.343 (1.746–6.403)	<0.001	3.409 (1.563–7.435)	0.002
ALB (g/L)				
>30	1			
≤30	1.016 (0.585-1.764)	0.954		
Cr (µmol/L)				
≤84	1			
>84	1.282 (0.730–2.253)	0.387		
hsCRP (mg/L)				
≤8	1			
>8	2.540 (1.338–4.821)	0.004		
C3/C4				
Normal	1			
Decrease	1.355 (0.715–2.571)	0.352		

^#^GCs pulse therapy, equivalent to methylprednisolone 500/1,000 mg/d.

^$^Current GCs dose, equivalent to prednisone.

^&^Including any one of the following immunosuppressants: cyclophosphamide, mycophenolate mofetil, methotrexate, azathioprine, leflunomide, cyclosporine A, and tacrolimus.

^※^Including any one of the following biological agents: rituximab and belimumab.

CMV, cytomegalovirus; DNA, deoxyribonucleic acid; SLE, systemic lupus erythematosus; SLEDAI, SLE disease activity index; GCs, glucocorticoids; ALT, alanine aminotransferase; ULN, upper normal limit; ALB, albumin; Cr, creatinine; hsCRP, hypersensitive C-reactive protein; C3/C4, complement C3/C4.

### Risk factors for 3-month all-cause mortality in SLE complicated by active CMV infection patients

Ten patients died within 3 months; all patients who died had CMV disease. The median time to death was 21.5 days (IQR 7.75–53). All patients who died were female, and the average age was 39 ± 10 years. All patients who died were treated with GCs within 1 month, of which four patients (4/10, 40.0%) received GCs pulse therapy. Nine patients (9/10, 90.0%) were treated with immunosuppressants within 3 months (including CTX in six patients, MMF in seven patients, CsA in one patient, and FK506 in one patient). Non-patients were treated with biological agents within 3 months.

**Figure 1:**
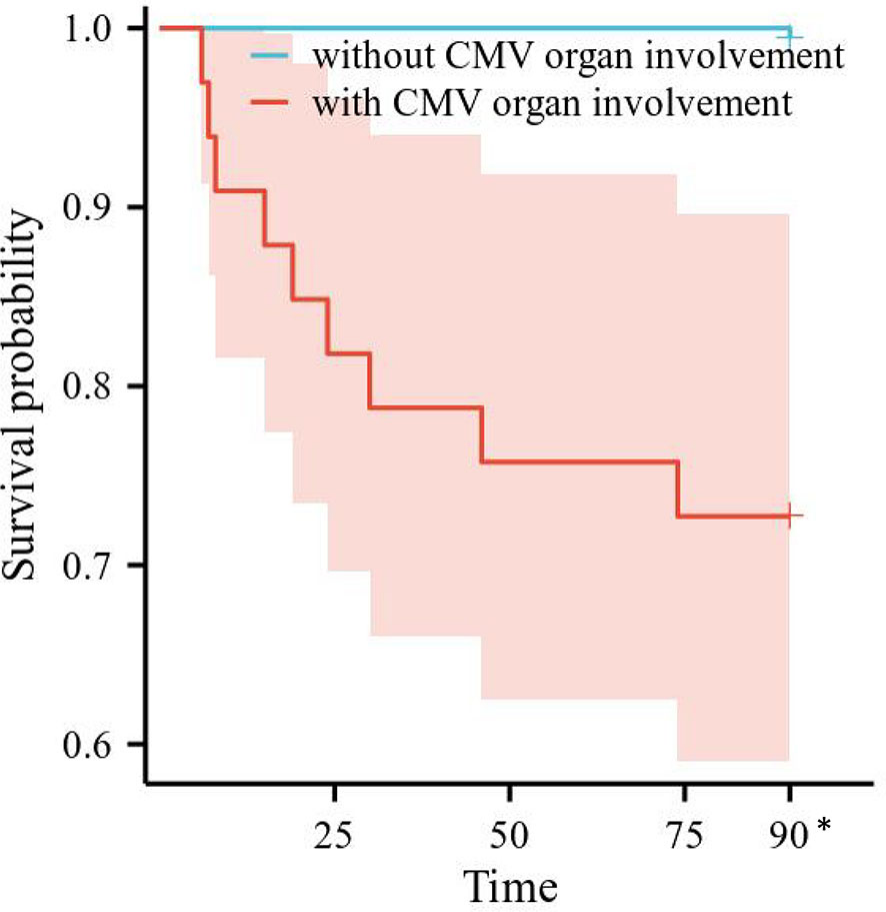
Survival curves of all-cause mortality in patients with or without CMV organ involvement. CMV, cytomegalovirus. *All the patients censored at this time point.

Risk factors for 3-month all-cause mortality were analyzed in the 206 SLE complicated by active CMV infection patients. Differences in survival by subgroup were tested with Cox regression, after testing that proportional hazard assumptions were satisfied. We conducted a univariate Cox regression analysis involving gender, age, SLE duration, SLEDAI-2000, plasma CMV DNA viral load, the use and dosage of medications, and laboratory examinations. The results are shown in **Table 5**. CMV organ involvement, plasma CMV DNA viral load, number of SLE involved system, lymphopenia, moderate to severe anemia, and hsCRP were included in a multivariable Cox regression model. The finding indicated that CMV organ involvement [hazard ratio (HR) 47.222, 95% CI 5.621–396.689, *p* < 0.001], number of SLE involved system (HR 1.794, 95% CI 1.029–3.128, *p* = 0.039), and elevated hsCRP (HR 5.767, 95% CI 1.190–27.943, *p* = 0.030) were independent risk factors for 3-month all-cause mortality. Survival curves of 3-month all-cause mortality in patients with or without CMV organ involvement are shown in **Figure 1**.

**Table 5:** Risk factors for 3-month all-cause mortality in SLE complicated by active CMV infection patients.

Variable	Univariate analysis		Multivariable analysis	
	Hazard ratio (95% CI)	*p-*value	Hazard ratio (95% CI)	*p-*value
Gender				
Female	1			
Male	0.000 (0.000–Inf)	0.998		
Age (years)	1.024 (0.983–1.067)	0.256		
SLE duration^a^ (months)	1.001 (0.994–1.008)	0.764		
CMV organ involvement				
No	1			
Yes	55.139 (6.979–435.638)	<0.001	47.222 (5.621–396.689)	< 0.001
CMV DNA				
Negative	1	0.058		
Positive	7.363 (0.933–58.121)			
Number of SLE involved system	1.562 (1.082–2.256)	0.017	1.794 (1.029–3.128)	0.039
SLEDAI-2000	1.009 (0.922–1.104)	0.853		
Use of GCs pulse therapy^#^ within 1 month				
No	1			
Yes	1.303 (0.368–4.619)	0.682		
Current GCs dose^$^ (mg/d)				
0–60	1			
≥60	1.659 (0.468–5.877)	0.433		
Use of immunosuppressant^&^ within 3 months				
No	1			
Yes	2.307 (0.292–18.207)	0.428		
Use of biological agents^※^ within 3 months				
No	1			
Yes	0.000 (0.000–Inf)	0.998		
WBC (10^9^/L)				
≥4	1			
<4	0.570 (0.121–2.686)	0.478		
LY (10^9^/L)				
≥0.5	1			
<0.5	9.793 (2.079–46.124)	0.004		
Hb (g/L)				
≥90	1			
<90	6.155 (1.307–28.989)	0.022		
PLT (10^9^/L)				
≥100	1			
<100	2.561 (0.741–8.847)	0.137		
ALT				
≤ULN	1			
>ULN	1.210 (0.313–4.680)	0.782		
ALB (g/L)				
>30	1			
≤30	2.509 (0.649–9.703)	0.183		
Cr (µmol/L)				
≤84	1			
>84	1.021 (0.288–3.617)	0.975		
hsCRP (mg/L)				
≤8	1		1	
>8	12.496 (2.653–58.864)	0.001	5.767 (1.190–27.943)	0.030
C3/C4				
Normal	1			
Decrease	0.503 (0.142–1.782)	0.287		

a SLE duration, the interval between initial SLE symptom onset and the current diagnosis of active CMV infection.

^#^GCs pulse therapy, equivalent to methylprednisolone 500/1,000 mg/d.

^$^Current GCs dose, equivalent to prednisone.

^&^Including any one of the following immunosuppressants: cyclophosphamide, mycophenolate mofetil, methotrexate, azathioprine, leflunomide, cyclosporine A, and tacrolimus.

^※^Including any one of the following biological agents: rituximab and belimumab.

CMV, cytomegalovirus; DNA, deoxyribonucleic acid; SLE, systemic lupus erythematosus; SLEDAI, SLE disease activity index; GCs, glucocorticoids; WBC, white blood cell; LY, lymphocyte; Hb, hemoglobin; PLT, platelet count; ALT, alanine aminotransferase; ULN, upper normal limit; ALB, albumin; Cr, creatinine; hsCRP, hypersensitive C-reactive protein; C3/C4, complement C3/C4.

### Risk factors for CMV recurrence within 3 months in SLE complicated by active CMV infection patients

A total of 12 patients experienced CMV recurrence within 3 months. The median time to recurrence was 53 days (IQR 15.5–67.5). All recurrent patients were female, and the average age was 37 ± 16 years. All recurrent patients were treated with GCs within 1 month before CMV reactivation, of which one patient (8.3%) received GCs pulse therapy. All recurrent patients were treated with immunosuppressants within 3 months (including CTX in seven patients, MMF in seven patients, CsA in three patients, and FK506 in two patients). None of the recurrent patients were treated with biological agents.

**Figure 2:**
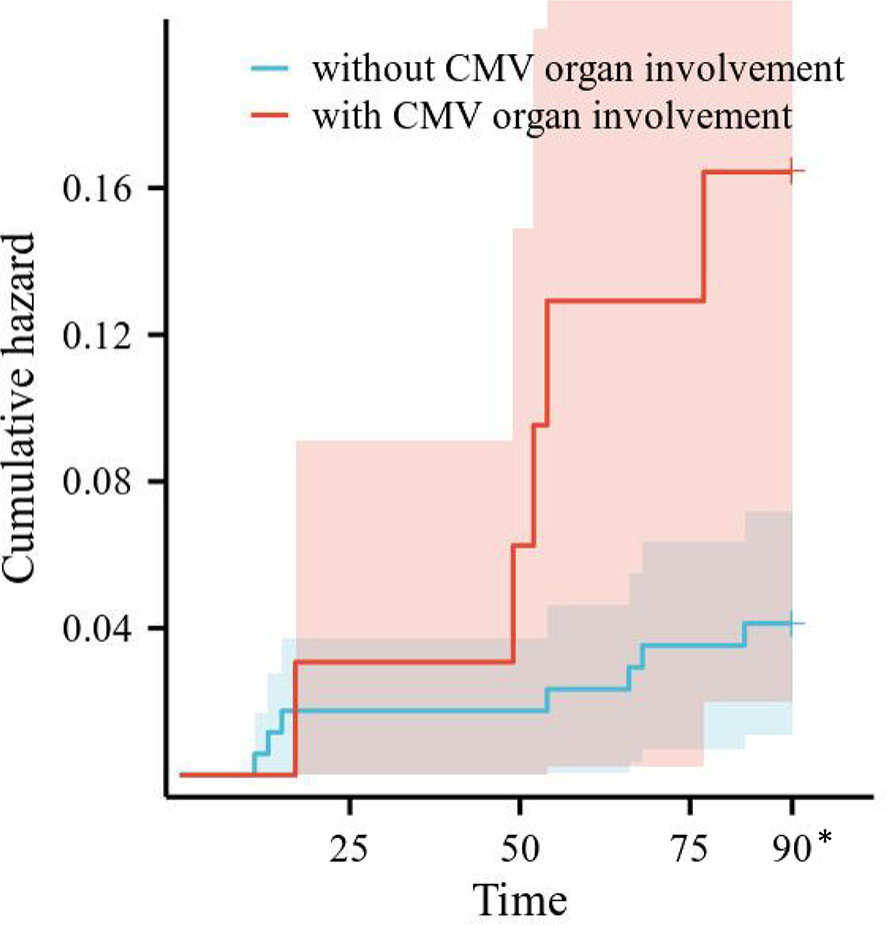
Survival curves of CMV recurrence in patients with or without CMV organ involvement. CMV, cytomegalovirus. *All the patients censored at this time point.

Risk factors for CMV recurrence within 3 months were analyzed in the 206 SLE complicated by active CMV infection patients. Differences in CMV recurrence by subgroup were tested with Cox regression, after testing that proportional hazard assumptions were satisfied. We conducted univariate Cox regression analysis on gender, age, SLE duration, SLEDAI-2000, plasma CMV DNA viral load, the use and dosage of medications, and laboratory examinations. The results are shown in **Table 6**. CMV organ involvement, plasma CMV DNA viral load, and C3/C4 were incorporated into a multivariable Cox regression model. The outcomes revealed that CMV organ involvement (HR 3.404, 95% CI 1.074–10.793, *p* = 0.037) independently posed a risk for CMV recurrence within 3 months. Survival curves of CMV recurrence in patients with or without CMV organ involvement are shown in **Figure 2**.

**Table 6:** Risk factors for CMV recurrence within 3 months in SLE complicated by active CMV infection patients.

Variable	Univariate analysis		Multivariable analysis	
	Hazard ratio (95% CI)	*p*-value	Hazard ratio (95% CI)	*p*-value
Gender				
Female	1			
Male	0.000 (0.000–Inf)	0.998		
Age (years)	1.016 (0.977–1.056)	0.428		
SLE duration^a^ (months)	1.002 (0.995–1.008)	0.593		
CMV organ involvement				
No	1		1	
Yes	3.943 (1.251–12.427)	0.019	3.404 (1.074–10.793)	0.037
CMV DNA				
Negative	1		1	
Positive	4.108 (0.900–18.749)	0.068	3.617 (0.786–16.638)	0.099
Number of SLE involved system	1.039 (0.709–1.523)	0.845		
SLEDAI-2000	1.014 (0.934–1.100)	0.745		
Use of GCs pulse therapy^#^ within 1 month				
No	1			
Yes	0.655 (0.177–2.418)	0.525		
Current GCs dose^$^ (mg/d)				
0–60	1			
≥60	1.562 (0.496–4.921)	0.447		
Use of immunosuppressant^&^ within 3 months				
No	1			
Yes	0.749 (0.203–2.767)	0.665		
Use of biological agents^※^ within 3 months				
No	1			
Yes	1.731 (0.224–13.410)	0.599		
WBC (10^9^/L)				
≥4	1			
<4	1.145 (0.345–3.801)	0.826		
LY (10^9^/L)				
≥0.5	1			
<0.5	1.154 (0.347–3.832)	0.815		
Hb (g/L)				
≥90	1			
<90	1.038 (0.330–3.272)	0.949		
PLT (10^9^/L)				
≥100	1			
<100	0.820 (0.222–3.028)	0.766		
ALT				
≤ULN	1			
>ULN	2.039 (0.647–6.423)	0.224		
ALB (g/L)				
>30	1			
≤30	0.524 (0.158–1.739)	0.291		
Cr (µmol/L)				
≤84	1			
>84	1.518 (0.490–4.707)	0.470		
hsCRP (mg/L)				
≤8	1			
>8	0.949 (0.257–3.504)	0.937		
C3/C4				
Normal	1			
Decrease	0.325 (0.105–1.007)	0.051		

a SLE duration, the interval between initial SLE symptom onset and the current diagnosis of active CMV infection.

^#^GCs pulse therapy, equivalent to methylprednisolone 500/1,000 mg/d.

^$^Current GCs dose, equivalent to prednisone.

^&^Including any one of the following immunosuppressants: cyclophosphamide, mycophenolate mofetil, methotrexate, azathioprine, leflunomide, cyclosporine A, and tacrolimus.

^※^Including any one of the following biological agents: rituximab and belimumab.

CMV, cytomegalovirus; DNA, deoxyribonucleic acid; ICU, intensive care unit; SLE, systemic lupus erythematosus; SLEDAI, SLE disease activity index; GCs, glucocorticoids; WBC, white blood cell; LY, lymphocyte; Hb, hemoglobin; PLT, platelet count; ALT, alanine aminotransferase; ULN, upper normal limit; ALB, albumin; Cr, creatinine; hsCRP, hypersensitive C-reactive protein; C3/C4, complement C3/C4.

## Discussion

In the present study, we conducted an exploration of the clinical features and prognosis in SLE patients complicated by active CMV infection. This will provide information on management strategies of CMV infection in the SLE population.

Few studies have explored the association and diagnostic value between plasma CMV DNA viral load and pp65 antigenemia in the SLE population. Inconsistent with previous findings in the transplant population (18, 19), a study compared the CMV DNA viral load and pp65 antigenemia test in the SLE patients for monitoring the development of CMV disease, and they found that pp65 antigenemia had a higher sensitivity (87%) but poor specificity (7.6%), CMV DNA viral load had a moderate sensitivity (66.1%) and specificity (55.3%), and there was no consistency between plasma CMV DNA viral load and pp65 antigenemia (Kappa = −0.176, *p* = 0.055) (19). In addition, a systematic review revealed that a high CMV DNA viral load was linked to the development of CMV disease in individuals with SLE (20). In this study, there was a lack of consistency between plasma CMV DNA viral load and pp65 antigenemia in the SLE population (Kappa < 0, *p* < 0.05), and plasma CMV DNA viral load seemed to have a higher value in diagnosing CMV disease. There may be cross-reactive antigens related to CMV pp65 in SLE patients (21, 22), thereby reducing the diagnostic value of pp65 antigenemia in CMV disease.

Several studies linked treatment drugs to CMV disease. Xue Y et al. reported that the CMV pneumonia group received higher doses of PSL [median (range) 32 (4–100) mg vs. 20 (1–50) mg/d, respectively, *p* < 0.010] and more frequently immunosuppressants (79% vs. 58%%*p* < 0.010) than the subclinical CMV infection group in the RD population (9). Another study of 38 SLE patients observed that the CMV disease group received a higher PSL dosage compared to the non-CMV disease group [mean (SD) 25.9 (17.1) mg/d vs. 9.0 (4.1) mg/d, respectively, *p* = 0.006], and the use of AZA 1 month prior to admission was more common in the CMV disease group (35% vs. 5.6%, *p* = 0.045) (23). The results of this study were partially similar to those of previous studies, which indicate that higher doses of GCs were associated with CMV disease (OR = 2.155, *p* = 0.031), while there were no significant differences in the GCs pulse therapy and immunosuppressant usage. The discrepancy may be due to different definition of CMV infection, types and activity of RD.

We proposed a certain level of positive association between CMV disease and SLE activity. Xue Y et al. found that patients with CMV pneumonia had a longer SLE duration compared to patients without CMV disease [median (range) 8 (0.03–360) months vs. 3 (0.25–156) months, respectively, *p* < 0.05] (9). Results of a clinical study in autoimmune disease patients (mostly SLE) complicated by active CMV infection showed that the deceased subgroup had a significantly higher SLEDAI-2000 than the alive subgroup (*p* = 0.072) (23). In the present study, number of SLE involved system was higher in the CMV disease group than in the subclinical CMV infection group, while there were no significant differences in the SLEDAI-2000 and SLE disease duration. In addition, SLE multi-system involvement was associated with 3-month all-cause mortality (HR = 1.794, *p* = 0.039). Although they are most widely used for the assessment of SLE activity, number of involved system and SLEDAI-2000 have some limitations, e.g., insufficient attention to the rare but important symptoms of SLE. The relationship between CMV disease and SLE activity should be confirmed in the future.

One study of 56 SLE patients with CMV diseases revealed a significant difference in the percentage of patients who had CMV end-organ diseases between the mortality and survival groups (83.33% vs. 25%, HR = 15.000, *p* = 0.001) (24). In a single-center-based nested case–control study, 113 patients who underwent haploidentical HSCT (2.92%) experienced CMV disease, and the overall mortality was higher in patients with CMV pneumonia, disseminated CMV disease, and CMV encephalitis (61.7%, 57.1%, and 40.0%, respectively) (25). In our study, CMV organ involvement accounted for 37.1% (33/89) of SLE complicated by CMV disease, with case fatality rates within 3 months of up to 27.3% (9/33); CMV organ involvement (HR = 47.222, *p* < 0.001) and elevated hsCRP, a well-known clinical biomarker for inflammation (HR = 5.767, *p* = 0.030), were independent risk factors for 3-month all-cause mortality in SLE patients complicated by active CMV infection.

Despite effective antiviral treatment, a proportion of patients may experience relapse. Risk factors for CMV recurrence are incompletely characterized; most studies have been conducted in transplant recipients. An investigation indicated that 19.4% (33/170) of solid organ transplant recipients encountered a relapse of CMV within 6 months, and low absolute lymphocyte count was an independent predictor for the recurrence of CMV disease (HR 1.11, *p* = 0.009) (26). Some scholars have proposed that high CMV DNA load at diagnosis was associated with risk of recurrence (27, 28). To our knowledge, our research is the first study to analyze risk factors for CMV recurrence in SLE patients. We found that the CMV recurrence rate within 3 months was only 5.8% in SLE patients, much lower than that in transplant recipients (19.4%–73%) (26, 29, 30). Notably, CMV organ involvement was an independent risk factor for CMV recurrence within 3 months (HR = 3.404, *p* = 0.037). The varying outcomes could be attributed to disparities in the study population, antiviral therapy, the time frame retrospectively examined, and so forth.

Threshold of plasma CMV DNA viral load or pp65 antigenemia for preemptive therapy may be different in different risk populations. Although low threshold is likely to be more clinically meaningful in patients with a higher infection risk, choosing a very low threshold may lead to unnecessary treatment. A systematic review revealed that the timing of preemptive therapy was not uniform across the studies (31), and several international guidelines recommend that medical institutions define thresholds for triggering therapy based on risk categories and center data (20, 32). In our study, of the 15 patients who did receive anti-CMV therapy, the median plasma viral load was 500 copies/mL, the median WBC count of peripheral blood with positive CMV pp65 antigen was 3, and none of the patients died or developed target organ invasion within 3 months. In a retrospective cohort analysis of non-immunocompromised patients with CMV reactivation, the use of ganciclovir did not show any significant connection with long-term outcomes; therefore, antiviral treatment in such cases may not be deemed necessary unless there are concerns of organ involvement (33). Studies on rheumatic disease patients complicated by active CMV infection also found that pp65 turned negative spontaneously in some patients (17, 34). Thus, antiviral therapy may not be necessary for some patients with subclinical CMV infection or even CMV syndrome. The relatively limited sample size of the untreated group emphasizes the need for more expansive studies in the future.

This study inevitably has some limitations. First, the enrolled patients were hospitalized at Peking Union Medical College Hospital; they seemed to be more seriously ill, and some patients were excluded because of grossly incomplete medical records; thus, there is a potential for selection bias in this study. Second, some factors such as viral replication kinetics and lymphocyte subsets cannot be further analyzed due to missing data. Third, because of the limited number of observed outcome events, the estimated incidences have broad confidence intervals, which necessitate further research for validation. Lastly, this study cannot establish a causal relationship, emphasizing the need for prospective investigations in the future.

## Conclusion

In summary, our study explored the clinical characteristics and outcomes of SLE complicated by active CMV infection. We found that peripheral blood CMV DNA viral load seemed to have a higher value in the diagnosis of CMV disease; patients with CMV organ involvement, SLE multi-system involvement, and elevated hsCRP might have a higher risk of 3-month all-cause mortality; and patients with CMV organ involvement might have a higher risk of CMV recurrence within 3 months. These findings contribute to CMV management targeting SLE patients.

## Data availability statement

The original contributions presented in the study are included in the article/supplementary material. Further inquiries can be directed to the corresponding authors.

## Ethics statement

The studies involving humans were approved by the Ethics Committee of Peking Union Medical College Hospital. The studies were conducted in accordance with the local legislation and institutional requirements. Written informed consent for participation was not required from the participants or the participants’ legal guardians/next of kin because this study did not involve contact with participants or any intervention.

## Author contributions

YC: Writing – original draft. LFZ: Data curation, Methodology, Writing – review & editing. YCL: Writing – review & editing. YL: Writing – review & editing. LDZ: Writing – review & editing. BZ: Writing – review & editing. GR: Writing – review & editing. XS: Funding acquisition, Writing – review & editing. XL: Resources, Writing – review & editing.
